# Evaluation of non-invasive prenatal testing (NIPT) for aneuploidy in an NHS setting: a reliable accurate prenatal non-invasive diagnosis (RAPID) protocol

**DOI:** 10.1186/1471-2393-14-229

**Published:** 2014-07-16

**Authors:** Melissa Hill, David Wright, Rebecca Daley, Celine Lewis, Fiona McKay, Sarah Mason, Nicholas Lench, Abigail Howarth, Christopher Boustred, Kitty Lo, Vincent Plagnol, Kevin Spencer, Jane Fisher, Mark Kroese, Stephen Morris, Lyn S Chitty

**Affiliations:** 1 North East Thames Regional Genetics Service, Great Ormond Street Hospital for Children NHS Foundation Trust, Level 5, York House, 37 Queen Square, London WC1N 3BH, UK; 2 Genetics and Genomic Medicine, UCL Institute of Child Health and Great Ormond Street Hospital for Children NHS Foundation Trust, London, UK; 3 Centre for Medical Statistics & Bioinformatics, Plymouth University, Plymouth, UK; 4 Fetal Medicine Unit, University College London Hospitals NHS Foundation Trust, London, UK; 5 UCL Genetics Institute, London, UK; 6 Clinical Biochemistry Department, Barking, Havering & Redbridge University Hospitals NHS Trust, Essex, UK; 7 Antenatal Results and Choices, London, UK; 8 PHG Foundation, Cambridge, UK; 9 Research Department of Applied Health Research, University College London, London, UK

**Keywords:** Non-invasive prenatal testing, Cell-free fetal DNA, Aneuploidy, Prenatal diagnosis

## Abstract

**Background:**

Non-invasive prenatal testing (NIPT) for aneuploidies is now available through commercial companies in many countries, including through private practice in the United Kingdom (UK). Thorough evaluation of service delivery requirements are needed to facilitate NIPT being offered more widely within state funded healthcare systems such as the UK’s National Health Service (NHS). Successful implementation will require the development of laboratory standards, consideration of stakeholder views, an analysis of costs and development of patient and health professional educational materials.

**Methods/Design:**

NIPT will be offered in an NHS setting as a contingent screening test. Pregnant woman will be recruited through six maternity units in England and Scotland. Women eligible for Down’s syndrome screening (DSS) will be informed about the study at the time of booking. Women that choose routine DSS will be offered NIPT if they have a screening risk ≥1:1000. NIPT results for trisomy 21, 18, 13 will be reported within 7–10 working days. Data on DSS, NIPT and invasive testing uptake, pregnancy outcomes and test efficacy will be collected. Additional data will be gathered though questionnaires to a) determine acceptability to patients and health professionals, b) evaluate patient and health professional education, c) assess informed choice in women accepting or declining testing and d) gauge family expenses. Qualitative interviews will also be conducted with a sub-set of participating women and health professionals.

**Discussion:**

The results of this study will make a significant contribution to policy decisions around the implementation of NIPT for aneuploidies within the UK NHS. The laboratory standards for testing and reporting, education materials and counselling strategies developed as part of the study are likely to underpin the introduction of NIPT into NHS practice.

**NIHR Portfolio Number:**

13865

## Background

In England, Scotland and Wales the National Screening Committee (NSC) recommends that Down’s syndrome screening (DSS) is offered to all pregnant women. The combined screening test (CST), which uses both fetal ultrasound and maternal serum biomarkers to generate a risk assessment, is performed between 10 and 14 + 1 weeks gestation and has a detection rate of around 85% and a false positive rate of 2.5% when the risk cut off is 1:150. The quadruple test, which uses only maternal serum biomarkers, is performed between 14 + 2 and 20 weeks gestation and has a detection rate of around 70% and a false positive rate of 3.5% when the risk cut off is 1:150. In current NHS practice women who have a screening risk of 1:150 or greater are offered invasive prenatal diagnosis (IPD), either chorionic villus sampling (CVS) from 11 weeks or amniocentesis from 15 weeks, that will give a definitive diagnosis of Down’s syndrome (trisomy 21) or one of the other common aneuploidies (trisomy 18 and trisomy 13). Other chromosomal abnormalities may be detected with IPD if full karyotyping or microarray analysis is performed.

As invasive tests carry a risk of miscarriage of around 0.5% [1], researchers have long been focused on developing an alternative, highly accurate non-invasive approach. The possibility of diagnosing aneuploidies with a maternal blood test emerged with the discovery of cell free fetal DNA (cffDNA) in 1997 [2], and in 2008 the first proof-of-principle studies were published showing that non-invasive prenatal testing (NIPT) for aneuploidy was possible using next generation sequencing (NGS) [3,4]. NGS technology has advanced rapidly and NIPT for Down’s syndrome now has detection rates above 99% and false positive rates of 0.1-1% [5-10]. Detection rates from validation studies are also high for trisomy 18 (>97%) [10-14] and trisomy 13 (up to 90%) [10,11,13] and continue to improve [15,16]. The small false positive rate means that NIPT is not considered fully diagnostic and IPD is recommended to confirm a positive NIPT result [17-19]. There may be further scope for using NIPT for identifying other chromosomal abnormalities as NGS has been reported to detect other chromosomal rearrangements and molecular karyotyping with NIPT now seems feasible, although the limits of detection and false positive rates are yet to be determined [20-22].

NIPT for Down’s syndrome was first launched through commercial providers in the USA and China/Hong Kong in 2011. These commercial tests are now also available in Europe, including through private practice in the UK, where samples are sent for testing in the USA or Hong Kong. However, NIPT is not yet being offered within a state funded healthcare system such as the NHS. The RAPID (Reliable, Accurate Prenatal, non-Invasive Diagnosis) research programme (RP-PG-10107-0707) was established to investigate all aspects of implementing non-invasive tests based on cffDNA into the NHS [23]. Before NIPT for aneuploidy can be implemented into routine clinical practice within the NHS, further information is required to determine where it fits in the care pathway, uptake in clinical practice, whether the rate of invasive testing will change, the costs for the NHS and for service users, and how best to educate women and health professionals. There is information available to address some of these questions based on hypothetical scenarios [24-31] and research reporting NIPT uptake and experience outside the UK is beginning to appear [32]. However, to get a true understanding of how NIPT for aneuploidy would work best within the NHS DSS programme, it is important to thoroughly evaluate NIPT within this setting. This will allow delivery of a comprehensive assessment that includes laboratory requirements, test performance, cost considerations and service user and provider needs, preferences and opinions.

There are several possible approaches for introducing NIPT into the current NHS DSS programme [24]. The three most likely are;

1. An alternative to IPD, where NIPT is only offered to women with a high risk result.

2. A first line screening test to replace the current DSS programme.

3. Contingent screening, where all women with a DSS risk above a pre-specified level are offered NIPT.

Using NIPT as an alternative to IPD in the current care pathway, would result in a slight decrease in the number of Down’s syndrome cases detected and, as discussed above, IPD would still be required for confirmation of abnormal results. Recent modelling of costs in an NHS setting has shown that if NIPT were used as a first line screening test that replaced the current DSS programme there would be benefits in terms of cases detected and a reduction miscarriages resulting from invasive tests. However, this approach would be much more expensive than current DSS, even if the cost of NIPT was very low [33]. Another disadvantage of this approach is that potential additional benefits conferred by current DSS would be lost, including early identification of other fetal abnormalities and pregnancies at risk of preeclampsia and intrauterine growth restriction (IUGR) [34]. Offering NIPT using a contingent approach will provide the same or better outcomes as current DSS in terms of cases detected, have fewer procedure-related miscarriages and be less costly than the current screening pathway [33]. Further, it would be possible to adjust screening cut-off levels in order to stay within the existing DSS budget if necessary. For these reasons, in this study NIPT will be included in the current DSS care pathway as a contingent screening test. We propose to offer NIPT to women who choose to have DSS and have a screening result of 1:1000 or greater. The cut-off of 1:1000 is expected to provide a good balance between the cost of the test, improving detection of Down’s syndrome cases and decreasing the number of invasive tests performed [24,33].

The benefits of contingent screening could be enhanced further if the accuracy of the initial DSS tests were improved as fewer NIPT tests would be needed. The current DSS tests include the measurement of two maternal serum biomarkers in the first trimester (PAPP-A and free-beta-hCG) and four in the second trimester (alphafeto-protein (AFP), inhibin-A, oestriol and free-beta-hCG or total hCG). Recently it has been reported that the addition of other biomarkers, for example AFP and placental growth factor (PlGF) to the first trimester test has the potential to improve the detection of Down’s syndrome pregnancies and may also help identify pregnancies at high risk of other adverse pregnancy outcomes (preeclampsia and intra-uterine fetal growth restriction) [35,36]. As part of this study we will measure additional maternal serum biomarkers, including AFP and PIGF, in women undergoing DSS at a subset of participating hospitals. These data will then be used with the currently measured markers to examine multiple algorithms for evaluating the risk of Down’s syndrome and explore whether or not these markers can accurately predict other adverse pregnancy outcomes.

Thorough evaluation of implementation strategies for NIPT requires approaches that not only examine laboratory performance and test uptake but also consider psychosocial issues, educational material and health economic data. While the prospect of a test that is available earlier in pregnancy and does not place the fetus at risk is welcomed by many potential service users [27,29,37], there are significant concerns around how this should happen in order to prevent the potential for testing to become routine and to maintain informed choice [38-41]. Furthermore, we have shown that there are significant differences in the values women and health professionals place on aspects of NIPT and how it should be offered [26]. There are also divergent views about some ethical aspects of delivery of NIPT, for example, the need for written consent and for time for reflection between information-giving and consenting to the test, and concerns over pressure to undergo testing if the risk of miscarriage is removed [27,41]. These aspects will be investigated in this study to enable us to make evidence-based recommendations about best practice.

Another significant consideration for implementation is cost. Currently NIPT is offered at £400-£750 in the UK, which includes the cost of sending samples overseas for analysis. Accurate and detailed costing under NHS clinical practice conditions is likely to be an important factor affecting widespread adoption of NIPT. In order to deliver a reliable economic evaluation, an accurate estimate of uptake of NIPT and the effect of introducing NIPT on the uptake of both DSS and any subsequent IPD is required. Work presenting hypothetical scenarios to women suggests that the uptake of DSS is likely to increase with the implementation of NIPT [27] and emerging evidence from offering the test in the USA shows fewer women turn down follow-up testing after a high risk screening result following the introduction of NIPT [32]. However, the impact of introducing NIPT into the NHS DSS programme will not be clear until evaluated in a ‘real-life’ situation.

## Aims and objectives

The overall aim of the study is to evaluate service delivery requirements for the implementation of NIPT for aneuploidies within the NHS.

### Principal objectives

•Identify the main barriers and facilitators for implementing NIPT within the NHS

•Collect data that could be used to evaluate NIPT in NHS clinical practice

•Evaluate patient uptake and experience of NIPT for Down’s syndrome

### Secondary objectives

•Collect data that could be used to improve current DSS and prediction of adverse pregnancy outcomes

•Pilot and evaluate health professional education materials

•Pilot and evaluate patient information materials

•Explore the acceptability of NIPT to service users including acceptability as a contingent screening test and as an assessment of informed choice

•Compare uptake of screening, NIPT and invasive testing. Including an evaluation of any differences related to ethnicity, parity, age and other routinely collected parameters

•Perform a detailed health economic evaluation

•Confirm the sensitivity and specificity of NIPT in an intermediate risk population

## Methods/Design

### Ethical approval

This study forms part of the RAPID programme which is evaluating standards for the implementation of non-invasive testing based on cffDNA in the UK NHS [23]. NHS Research Ethics Committee approval for the study has been obtained (13/LO/0082).

### Study design

**Figure 1:**
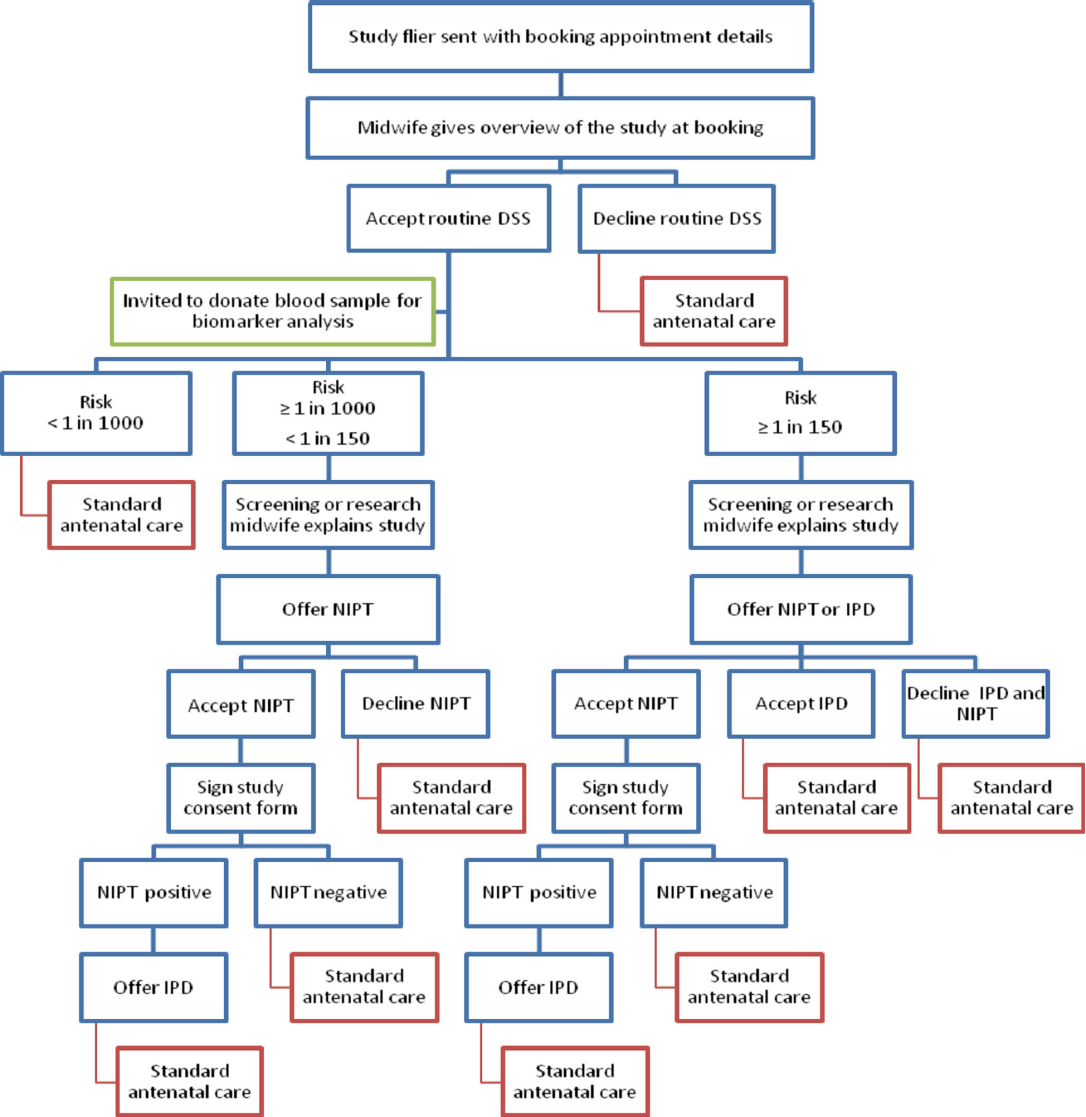
Overview of study protocol and the relationship with the clinical care pathway.

This is a multi-centre observational study. Figure 1 provides an overview of how the study fits within the current clinical care pathway.

### Population

Women will be recruited from 6 maternity units in England and Scotland, which together have around 32500 births annually. The selected maternity units include District General and Teaching Hospitals, in both urban and rural areas and encompass a wide ethnic and social mix. In addition there is a wide variation in uptake of current DSS at these trusts (56.5-93%) and IPD in screen positive women (50–85.4%). The pathway for delivering DSS varies and includes one-stop clinics and two-stage pathways which include scanning and blood draw performed at one appointment followed by a phone call on another day with results.

### Sample size

The overall study sample size was justified on the precision achieved for the differences in overall detection rate and false positive rate between the existing DSS pathway and the new contingent NIPT pathway. A screened sample of 25,000 will enable the reduction in false positive rate with the contingent pathway compared to the existing pathway to be estimated to within ±0.2%. With an anticipated reduction of 2.4% or higher, this corresponds to a 95% confidence interval extending from 2.2% to 2.6%. This sample size will enable us to estimate the increase in detection rate to within ±0.65%. With an anticipated increase in detection rate of at least 8%, this corresponds to a 95% confidence interval ranging from 1.5% to 14.5%, thus enabling us to confirm the increased detection rate with the contingent pathway. Although not all women take up DSS, recruitment in 6 units with total annually bookings of more than 32,500 will allow us to achieve the required sample size.

This total population and number of units is also required to allow us to evaluate change in the uptake of both screening and diagnostic testing so as to get a robust estimate of change that will inform a detailed economic analysis.

The biomarker analysis will be conducted in three units with combined annual bookings of approximately 20000. The potential sample size of 18,000 pregnancies (assuming not all women accept DSS) has sufficient power to detect an effect given the prevalence of severe preeclampsia of around 1:200 pregnancies.

### Inclusion criteria

Inclusion criteria for this study are: pregnant women who book before 20 weeks gestation who accept DSS as part of routine care; older than 16 years; able to read and understand English and provide informed consent; non-English speakers where there is a trained interpreter available as part of the routine clinical service.

### Exclusion criteria

Exclusion criteria for this study are: women booking after 20 weeks; younger than 16 years; women who declined DSS; women with multiple pregnancies and those who are unable to understand the participant information sheet because English is not their first language.

### Recruitment

Prior to the start of the study an education package for health professionals that includes face-to-face training and written information will be delivered at each participating maternity unit. Every pregnant woman who books at a participating hospital and is eligible for DSS will be informed about the study through a participant information sheet sent with her booking appointment details and brief discussion of the study with the midwife during her booking appointment. Women who choose to take up DSS will have the standard DSS test appropriate for their gestation. At three of the participating hospitals, women having DSS will be asked for their permission to use the excess serum for research to evaluate potential improvements in the current DSS test. The results of the extra marker analysis will not be reported in the current pregnancy, but will be used for modelling of new approaches for estimating risk. At all participating hospitals, women with a risk of 1:1000 or greater for trisomy 21, 18 or 13 will be offered the opportunity to discuss options for further testing. Standard local protocols for delivering DSS results will be followed and consent for participation in the study obtained. If the risk is 1:1000 to 1:151, the midwife will explain the DSS result and the study, check understanding and, if consent is given, take blood for NIPT. If the risk is ≥1:150, the midwife will discuss IPD, as is current standard practice at this risk, and NIPT including the benefits and limitations of both tests. If consent is given, women in this risk category can choose to have invasive testing or NIPT, but for those choosing IPD we will also seek consent for NIPT.

### Delivery of NIPT results

NIPT results for trisomy 21, 18 and 13 will be reported. The samples from women who also had an ultrasound abnormality or NT measurement of ≥3.5 mm will also receive a result for Turner syndrome (Monosomy X). Women will be phoned with their NIPT results as soon as available, and at most within 7–10 working days of taking blood.

### Predicted to be affected

•Women will be advised that whilst the likelihood is high that the result is correct (99%), as there is a small false positive rate (<1:100), IPD for confirmation is recommended.

### Highly unlikely to be affected

•For women with a DSS result of 1:1000 to 1:151, the midwife will explain that this is reassuring and confirms her low risk result (1:10000).

•For women with a DSS result of ≥1:150 who have not already had an invasive test, the midwife will explain that this is a reassuring result as data indicate that the NIPT test is >99% accurate, but offer IPD for confirmation should the woman wish it.

### Inconclusive NIPT result

•Women with a DSS result of 1:1000 to 1:151 will be told that as this is a new test we occasionally get an ‘unclear’ result (up to 4% of cases). She will be offered a repeat NIPT test.

•Women with a DSS result of ≥1:150 who have not already had IPD, will be offered repeat NIPT or IPD.

### Biomarker analysis

Up to 1 ml of excess serum from the standard DSS sample will be used for biomarker analysis at the clinical biochemistry laboratory at Barking, Havering & Redbridge University Hospitals NHS Trust, using standard immunoassay procedures. These data will be used with currently measured markers to explore multiple algorithms for the evaluation of risk of Down’s syndrome, but will also be correlated with other adverse pregnancy outcomes to determine whether or not these markers accurately predict other adverse pregnancy outcomes.

### NIPT testing

Maternal blood will be taken into EDTA tubes or, when there will be delay of more than 8 hours in transferring samples, into cell free DNA (cfDNA) blood collection tubes and sent to the laboratory. Blood will be centrifuged and cfDNA extracted from maternal plasma using the QIAsymphony (Qiagen, Netherlands) in the North East Thames Regional Genetics (NETRGL). Any remaining plasma and the blood cell pellets will be stored at −80°C for quality assurance purposes and further analysis until the pregnancy outcome is known.

Pooled patient libraries will be prepared and subjected to NGS (Illumina HiSeq 2500) at NETRGL using established protocols. Bioinformatic analysis of NGS data will be conducted using the RAPIDR package (http://cran.r-project.org/web/packages/RAPIDR/), developed in-house [42] and a result assigned to each patient. NGS libraries will be stored at −20°C until the pregnancy outcome is known for future use if repeat NGS testing is required.

### NIPT Validation

To provide benchmarking of NIPT performed in a CPA accredited lab (GOSH) we are collaborating with a commercial provider, Illumina, with experience of several thousand tests. Four hundred samples will be sent for testing with the Verifi® prenatal test [43], which detects the major trisomies and sex chromosome abnormalities if requested. If there is a discrepancy in the results from the NHS and Illumina laboratories, the result will be treated as “inconclusive” and the participant will be offered a repeat NIPT test or an invasive test as appropriate.

### Prenatal data collection and analysis

Uptake of DSS and invasive testing in women with DSS results ≥1:150 will be evaluated at all participating sites prior to offering NIPT. Following the introduction of NIPT, data on uptake of DSS, NIPT and IPD and NHS costs will be collected. Prenatal data collected will include routinely collected demographics and pregnancy details, including DSS results with absolute values and MoMs, crown-rump length, nuchal translucency measurement. Results of any IPD tests (qfPCR, full karyotype or microarray) will be obtained. In the event of pregnancy loss without IPD, results of any cytogenetic analysis of products of conception, placenta or skin will be obtained. We will collect pregnancy outcome data for all participants, this will include liaison with the local cytogenetics laboratories and the National Down’s Syndrome Cytogenetic Register to determine all cases of aneuploidy reported but not detected by NIPT in our cohort.

The characteristics of the study sample, including uptake, will be summarised graphically and in tables. Point and interval estimates (95% confidence intervals) will be produced for the following;

1. The overall and age-specific detection rates (DR) and false positive rates (FPR) of the combined test with risk cut-off of 1:150

2. Overall and age-specific rates of continuation (risks of 1:1000 or higher) to NIPT

3. The DR and FPR for contingent policies in which women with risks in of 1:1000 or higher are given NIPT

4. The differences in DR and FPR from between (1) and (3)

5. Change in uptake rates for DSS and IPD

Confidence intervals will be obtained using bootstrapping. The bootstrap samples will also be used to provide interval estimates for the health economic assessment. Analysis will be conducted using the R statistical software. This will be pre-specified in a detailed Statistical Analysis Plan (SAP) containing listings of all programmes that will be validated and reviewed independently.

We will also compare the uptake of DSS, NIPT and IPD and evaluate any differences related to ethnicity, parity, age and other routinely collected characteristics.

### Health economic evaluation

The economic analysis will assess the cost implications of implementing NIPT, including accounting for changes in uptake compared with the current system.

We will:

1. Delineate the two pathways for DSS: (1) the current pathway; and (2) a new pathway using NIPT as contingent screening.

2. Use data collected from local study sites to plot movement of pregnant women through the pathways, including accounting for differences in uptake. Data include uptake of screening, ultrasound scans, counselling, laboratory tests, NIPT uptake, number of diagnostic tests and pregnancy outcomes. Of particular importance is the avoidance of IPD, which will be accounted for in the analysis.

3. Identify unit costs associated with the main cost components of the diagnosis pathways from routine published sources and market prices, including the costs of NIPT.

4. Investigate the mean cost per pregnancy of the current and new DSS pathway including contingent NIPT.

5. Investigate the cost impact of reorganising laboratory services away from IPD to NIPT, including capital investment and staff retraining costs.

6. Analyse the expected budget impact to the NHS of implementing NIPT as contingent screening versus the current screening pathway based on the mean incremental costs per woman tested and the expected number of women tested.

The perspective of the analysis will be the NHS. While incremental personal and social services costs are commonly included in economic evaluations these are likely to be zero in this study and so will be omitted. The time horizon for the analysis is the time period from the start of pregnancy until the end of pregnancy. The units of analysis are the pregnancy, eg, we will calculate the mean cost per pregnancy using NIPT.

To investigate additional costs to families we will ask a subset of women (approximately N = 400) before and after the introduction of NIPT to complete a short questionnaire to ascertain how their use of NHS and other services has been affected by the implementation of NIPT. The questionnaire will ask women to report contacts with the charitable and private sectors, time off work for themselves or their families, and out-of-pocket expenses. The women approached to complete a questionnaire both before and after the introduction of NIPT will comprise: (1) women who declined DSS; (2) women whose DSS result was <1:1000; (3) women whose DSS result was between 1:1000–151; and (4) women with a risk ≥1:150.

### Evaluation of health professional education material

A face-to-face training session and information sheets have been developed to educate health professionals involved in discussing and offering NIPT. This approach will be evaluated and the results used to develop other teaching materials, available via e-learning and mobile apps. Training will be delivered by the research team in conjunction with Antenatal Results and Choices (ARC), a patient charity experienced in training health professionals in prenatal counselling. Two to three group training sessions will be offered in each maternity unit, with a subsequent cascade approach used to ensure complete coverage. This cascade approach is recommended by the NSC and widely used in England for delivering antenatal screening education.

Educational sessions will be evaluated using a multi-stage approach, comprising three surveys:

1. Pre-course survey – including demographics, confidence in discussing and offering NIPT, self-perceived knowledge of NIPT.

2. Immediate post-course survey - learners’ perceptions of the session with open questions exploring useful aspects and any suggested changes or improvements.

3. A post-course survey to explore knowledge and experience of NIPT.

In addition, focus groups or interviews will be conducted with approximately 20 course attendees to determine any impact on clinical practice and areas of unmet need that should be addressed in future courses.

### Evaluation of patient information

A patient information leaflet describing NIPT for aneuploidy has been developed based on previous work in RAPID [44]. Action research methodology will be used to evaluate the patient information leaflet with a short questionnaire. Results will be used to refine and improve the patient information.

### Evaluation of stakeholder views

A mixed methods approach will be adopted to evaluate stakeholder attitudes towards implementation of NIPT to elicit potential barriers to and facilitators for implementation.

1. Women who have been offered NIPT

To explore the views and experiences of women who have been offered NIPT as part of the study a mixed methods study including a questionnaire and interviews at two time points will be conducted. Time 1 (T1) = after accepting (or declining NIPT), after blood has been taken but prior to receiving test results; Time 2 (T2) = one month following receipt of test results. All women who accept or decline NIPT will be invited to complete both questionnaires. A subset will also be invited to take part in a telephone or face-to-face interview.

The T1 questionnaire comprises a number of validated psychological measures; the Multi-dimensional Model of Informed Choice (MMIC) [45], the Deliberation scale [46], the Decisional Conflict scale [47,48], and the State-Trait Anxiety Inventory [49]. Questions addressing their reasons for accepting or declining NIPT and a number of demographic questions will also be included. The T2 questionnaire includes the Decisional Regret scale [50] and the State-Trait Anxiety Inventory [49]. Questions addressing the outcome of their NIPT test and resulting actions will also be included.

At T1 a subset of women with varying demographic backgrounds, previous experiences of pregnancy and screening risk results will be invited to take part in an interview to explore knowledge and attitudes towards NIPT, factors influencing decision-making, motivation for testing and the decision-making process including deliberation, pre-test counselling and support. At T2 a similarly diverse subset of women, including women with positive and negative NIPT outcomes, will be invited to take part in an interview to reflect on their decision, explore their views on NIPT, discuss service delivery and their overall experience of taking part in the evaluation study.

2. Health professionals working in participating maternity units and other professionals, including healthcare managers and laboratory staff.

Health professionals from participating units will be invited to take part in qualitative interviews to discuss their experience of offering NIPT as part of their clinical service. Other stakeholders will be invited to explore their views on introducing NIPT into an NHS clinical service.

For all interviews we will follow the accepted approach for recruiting in qualitative research whereby interviews will continue until no new codes or themes are emerging and saturation is reached [51]. From previous experience we would anticipate that this is generally 20–40 interviews [44,52,53]. Interviews will be conducted in person or by phone, will last approximately 30–45 minutes, and be audiotaped and transcribed verbatim. To maintain confidentiality, participants will be assigned a pseudonym. Interview data will be analysed using thematic analysis [51].

Ultimately this arm of the study will be used to inform the further development of the patient and health professional information materials, public education and development of implementation strategies.

## Discussion

The overarching goal of this study is to evaluate NIPT for aneuploidy in an NHS setting. This will allow us to provide the NSC with data on the uptake of DSS, NIPT and IPD, NHS and patient costs and acceptability to service users and providers. This data can then inform NSC in its considerations on whether NIPT should to be included in the DSS programme. Hence, the main potential outcome of this research is that it will have instigated and supported the implementation of NIPT as contingent screening in the NHS. If this happens, it will result in safer prenatal testing for aneuploidies by reducing the number of invasive tests required. Importantly it will also serve to negate the current inequality of access that results from NIPT only being available privately. The laboratory standards for testing and reporting, education materials and guidelines for best practice developed as part of the study will underpin the implementation of NIPT into NHS services.

## Abbreviations

cffDNA: Cell-free fetal DNA; cfDNA: Cell free DNA; CST: Combined screening test; DSS: Down’s syndrome screening; NIPT: Non-invasive prenatal testing; RAPID: Reliable accurate prenatal non-invasive diagnosis.

## Competing interests

The authors declare that they have no competing interests.

## Authors’ contributions

All authors have contributed to the development of the study protocol and have approved the final version of the study protocol submitted for publication.

## Pre-publication history

The pre-publication history for this paper can be accessed here:

http://www.biomedcentral.com/1471-2393/14/229/prepub
